# Enhanced Cycle Stability of Zinc Sulfide Anode for High-Performance Lithium-Ion Storage: Effect of Conductive Hybrid Matrix on Active ZnS

**DOI:** 10.3390/nano9091221

**Published:** 2019-08-29

**Authors:** Quoc Hanh Nguyen, Taehyun Park, Jaehyun Hur

**Affiliations:** Department of Chemical and Biological Engineering, Gachon University, Seongnam-si, Gyeonggi-do 13120, Korea

**Keywords:** zinc sulfide, titanium carbide, anode, high-energy ball milling, Li-ion batteries

## Abstract

Zinc sulfide (ZnS) nanocrystallites embedded in a conductive hybrid matrix of titanium carbide and carbon, are successfully fabricated via a facile high-energy ball-milling (HEBM) process. The structural and morphological analyses of the ZnS-TiC-C nanocomposites reveal that ZnS and TiC nanocrystallites are homogeneously distributed in an amorphous carbon matrix. Compared with ZnS-C and ZnS composites, the ZnS-TiC-C nanocomposite exhibits significantly improved electrochemical performance, delivering a highly reversible specific capacity (613 mA h g^−1^ over 600 cycles at 0.1 A g^−1^, i.e., ~85% capacity retention), excellent long-term cyclic performance (545 mA h g^−1^ and 467 mA h g^−1^ at 0.5 A g^−1^ and 1 A g^−1^, respectively, after 600 cycles), and good rate capability at 10 A g^−1^ (69% capacity retention at 0.1 A g^−1^). The electrochemical performance is significantly improved, primarily owing to the presence of conductive hybrid matrix of titanium carbide and amorphous carbon in the ZnS-TiC-C nanocomposites. The matrix not only provides high conductivity but also acts as a mechanical buffering matrix preventing huge volume changes during prolonged cycling. The lithiation/delithiation mechanisms of the ZnS-TiC-C electrodes are examined via ex situ X-ray diffraction (XRD) analysis. Furthermore, to investigate the practical application of the ZnS-TiC-C nanocomposite, a coin-type full cell consisting of a ZnS-TiC-C anode and a LiFePO_4_–graphite cathode is assembled and characterized. The cell exhibits excellent cyclic stability up to 200 cycles and a good rate performance. This study clearly demonstrates that the ZnS-TiC-C nanocomposite can be a promising negative electrode material for the next-generation lithium-ion batteries.

## 1. Introduction

Rechargeable Li-ion batteries (LIBs) have been widely used for mobile portable electronic devices and electric vehicles, owing to their high energy densities, long lifecycles, and low self-discharge rates [1,2,3]. The rapidly increasing demand for electric vehicles and portable devices necessitates new alternative electrode materials with higher capacities, higher energy densities, and good safety. Currently, the most commonly used anode for LIBs is graphite, but it is unable to satisfy the demands of high energy and power densities, owing to its poor rate capacity, low tap density (<1 g cm^−3^), and critical safety concerns related to Li plating [4,5,6]. Hence, enormous efforts have been devoted to the development of high-performance anodes with high capacity and enhanced safety characteristics [7,8,9,10]. To satisfy these requirements, numerous Li-alloying anodes have been proposed as potential candidates for replacing graphite, including Li-Sn, Li-Si, Li-Sb, Li-P, and Li-Ge [11,12,13,14,15,16].

Metallic Zn is one of the promising alternative anodes for rechargeable LIBs owing to its high theoretical volumetric capacity (1511 mA h cm^−3^) and it can easily form various Zn-metal binary alloying compounds, such as Zn-Sb, Zn-P, Zn-Se, Zn-Te, and Zn-S, among others [17,18,19,20,21,22,23,24,25]. Additionally, its beneficial features including natural abundance, inexpensiveness, and environmental friendliness suggest Zn as a promising material for LIB anodes. Despite these merits, Zn-based anodes inevitably undergo large volume expansion (about 70% and 228% for Zn and ZnO anodes, respectively) [26,27] during Li alloying/dealloying, resulting in the particle pulverization of active particles upon extended electrochemical cycling, which lead to poor cycling performance, similar to other Li-alloying materials [12,16]. In this regard, the introduction of a conductive TiC-C hybrid matrix composed of titanium carbide (TiC) and amorphous C, which can serve as an efficient buffering matrix that improves the cyclic stability and rate capability, has been proved as a promising candidate for satisfying these requirements [28,29,30,31,32,33,34,35]. TiC can effectively suppress the internal stress and prevent the agglomeration of active particles upon extended cycling, owing to its good mechanical properties and electrochemical stability [36,37]. Moreover, the synergistic effect between titanium carbide and amorphous carbon in the conductive TiC-C hybrid matrix not only provides the high electrical conductivity but also mitigates particle aggregation, improving the electrochemical performance [33].

Another effective strategy for solving these problems is to create a metallic chalcogenide compound that can progressively react with Li-ions during cycling. In this respect, S is one of the potential candidates for LIB anodes owing to its cheapness, natural abundance, eco-friendliness, and very high theoretical specific capacity (1675 mA h g^−1^), which is much higher than other chalcogens (e.g., Se (678 mA h g^−1^) and Te (420 mA h g^−1^)) [38,39,40,41]. Unfortunately, the application of the standalone S-based electrode is impractical because of its low electronic conductivity (~5 × 10^−22^ S cm^−1^), the dissolution of Li polysulfides, and the large volume expansion upon extended cycling. Therefore, many ZnS-based composites have been reported as high-performance anode materials owing to their very high theoretical specific capacities (829 mA h g^−1^), but their electrochemical performance remains unsatisfactory, as shown in Table S1 [42,43,44,45,46,47,48,49]. Moreover, the lithiation/delithiation potential of an active ZnS anode is much higher than the reaction potential of metallic Li, preventing the safety concerns associated with Li plating, which eventually results in dendrite growth and short circuiting [50,51].

Herein, we report an effective strategy to obtain improved electrochemical performance of a ZnS-based nanocomposite, by introducing ZnS nanoparticles into an efficient matrix of TiC and amorphous C via a facile and scalable high-energy ball-milling (HEBM) process. The ZnS-TiC-C nanocomposite has the following advantages as a new anode for LIBs. (i) The reaction between Zn and S forms an II–VI ZnS compound, which mitigates the large volume expansion due to the progressive electrochemical reactions and contributes to a high capacity. (ii) The TiC and C hybrid matrix acts as a buffering matrix that can prevent particle agglomeration and suppress the high volume change of active ZnS particles during cycling. (iii) The high conductivity of the TiC-C hybrid matrix provides facile electron transport, thereby contributing to the excellent electrochemical performance of the ZnS-TiC-C electrodes. We demonstrate the practical application of the ZnS-TiC-C nanocomposite as a high-performance anode material in a full-cell investigation.

## 2. Materials and Methods

### 2.1. Material Preparation

The ZnS-TiC-C nanocomposites were synthesized through a facile HEBM process using a planetary ball milling (Pulverisette 5, Fritsch) machine. First, a mixture of zinc (≥98%, <10 µm, Sigma-Aldrich, St. Louis, MO, USA), sulfur (99.998%, trace metal basis, Sigma-Aldrich), and titanium (99.99%, 325 mesh, Alfa Aesar, MA, USA) powders was manually ground and then placed into a hardened zirconium oxide bowl (80 cm^3^) with hardened zirconium oxide balls (with different sizes of 3/8 and 3/16 inches) and tightly closed in a glovebox under an Ar atmosphere. The mass ratio of ZrO_2_ balls to powder was 20:1 and the total weight of mixing powders was 2.0 g. The first HEBM process was carried out at a rotation speed of 300 rpm at ambient temperature for 10 h. The resulting mixture was mixed and manually ground with an appropriate amount of acetylene carbon black powder (100% compressed, 99.9%, Alfa Aesar). The second HEBM process was then operated under the identical conditions that were applied in the first process, in order to form the conductive TiC phase in the resulting composites. For comparison, the same procedure was applied without Ti at the initial mixing stage to form ZnS-C with a ZnS:C weight ratio of 8:2. Another control sample of a ZnS composite was prepared similarly without Ti and C. Additionally, we determined the optimum content of the active material (ZnS) by changing the weight percentage of ZnS (from 60% to 90%), along with the corresponding amounts of Ti and amorphous C, such that the mass ratio of TiC:C was fixed at 1:2. These samples were denoted as ZnS(60%)-TiC-C, ZnS(70%)-TiC-C, ZnS(80%)–TiC-C, and ZnS(90%)-TiC-C.

### 2.2. Material Characterization

The crystal structures of the ZnS-based composites were analyzed with an X-ray diffractometer with Cu Kα radiation (λ = 0.15406 nm) in a 2θ range of 20–80° at a scan rate of 2° min^−1^ (XRD, D/MAX-2200 Rigaku, Japan, at the Smart Materials Research Center for IoT (internet of things) at Gachon University for its instrumental support). The ex-situ XRD measurements were conducted at three states (pristine, fully discharged, and fully charged) to investigate the reaction mechanism of the ZnS-TiC-C electrode. The electrochemical coin-type cells were disassembled using wolf looping pliers in a glove box and rinsed with anhydrous dimethyl carbonate. These dismantled electrodes were then dried and covered with a layer of Kapton film to prevent direct contact with air. Scanning electron microscopy (SEM, Hitachi S4700, Tokyo, Japan), high resolution transmission electron microscopy (HRTEM, TECNAI G2F30, FEI corp., OR, USA), and scanning transmission electron microscopy (STEM, TECNAI G2F30, FEI corp., OR, USA) with energy dispersive X-ray spectroscopy (EDS) were conducted to investigate the morphologies, compositions, and elemental distributions of the as-prepared samples. The morphologies of the ZnS-TiC-C and ZnS-C electrodes after 50 cycles were observed by ex-situ SEM analysis.

### 2.3. Electrochemical Measurements

To investigate the electrochemical performances, the working anodes were prepared by coating the slurries containing 70 wt% of active materials (the as-synthesized ZnS-TiC-C, ZnS-C, and ZnS), 15 wt% of conductive carbon black (Super P), and 15 wt% binder of polyvinylidene fluoride (PVDF) dissolved in *N*-methyl-pyrrolidone (NMP) solvent. The slurry coating onto Cu foils was performed by doctor blading, followed by drying at 70 °C overnight under vacuum. The dried electrodes were cut in the shape of circular discs with a diameter of 12 mm. The working electrodes were used as anodes for CR2032-type coin cells and assembled in an Ar-filled glovebox to avoid O_2_ and moisture. Metallic Li foil, 1 M LiPF_6_ dissolved in diethyl carbonate/ethylene carbonate (1:1 by *v*/*v*), and polyethylene were used as the counter electrode, electrolyte solution, and separator, respectively. The mass loadings of active materials and the electrodes were typically ~0.8 mg and ~1.2 mg, respectively. The volume of liquid electrolyte loaded in the cell was ~0.2 mL. Galvanostatic charge/discharge experiments were conducted at a constant current density of 0.1 A g^−1^ (~1/6 C-rate) within the potential range 0.01–2.5 V (vs. Li/Li^+^) using a battery cycler (WBCS3000, WonATech, Seoul, South Korea). The cyclic voltammogram (CV) was measured using a ZIVE MP1 (WonATech) instrument in the same potential range, at the scan rate of 0.1 mV s^−1^. The rate capabilities were measured at various current densities ranging from 0.1–10 A g^−1^ using a WBCS3000 (WonATech) battery cycler. A ZIVE MP1 (WonATech) analyzer was used for electrochemical impedance spectroscopy (EIS) measurements in the range of 100 kHz–100 mHz with a 10-mV alternating-current amplitude. For a full-cell investigation, as-prepared ZnS(80%)-TiC-C and LiFePO_4_-graphite (denoted as LFP-G) electrodes were used as an anode and cathode, respectively. The electrolyte, separator, and coin cell for the full cell were the same as those used in the half-cell. The LFP-G cathode was synthesized by mixing LiFePO_4_ (<5 µm, >97%, Sigma-Aldrich, St. Louis, MO, USA) and graphite (200 mesh, 99.9999%, Alfa Aesar) powders at a mass ratio of 7:3. The resulting mixture was ball-milled for 10 h at a rotation speed of 300 rpm in an Ar atmosphere. The slurry for the cathode material was prepared by mixing LFP-G (80%), Super P (10%), and PVDF (10%) dissolved in an NMP solvent. Then, the cathode slurry was coated onto an Al foil and dried at 70 °C overnight under vacuum. For testing the electrochemical performance of the full cell, CR2032 coin cells containing the cathode and anode were electrochemically pre-charged and pre-discharged to make the batteries more stable by removing the effects of solid–electrolyte interphase (SEI) layer formation. Then, these test cells were assembled in a glovebox. Galvanostatic cycling and rate capability tests were performed within the voltage range of 1.0–3.8 V using a battery cycler (WBCS3000, WonATech). The total capacity of the full cell was calculated according to the total mass of active cathode and anode materials, the mass ratio of the cathode to the anode was ~2:1.

## 3. Results and Discussion

**Figure 1:**
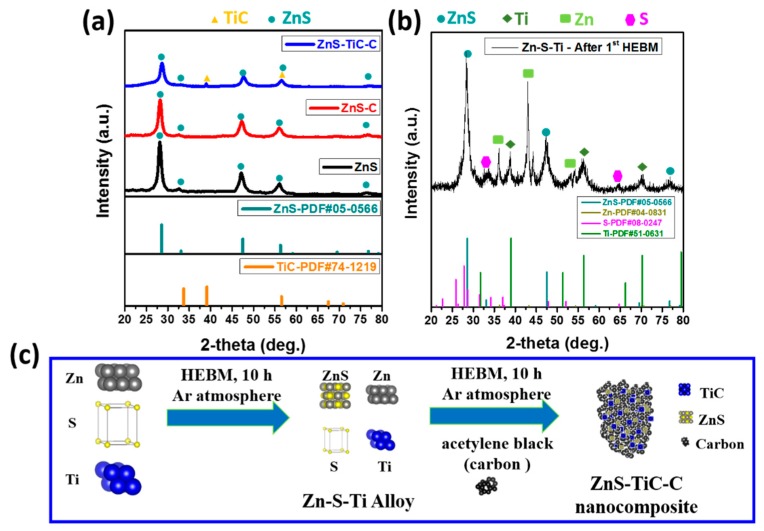
XRD patterns for (**a**) the ZnS, ZnS-C, and ZnS-TiC-C composites, (**b**) the Zn-S-Ti alloy after the first high-energy ball-milling (HEBM) process; and (**c**) schematic of the synthesis of the ZnS-TiC-C composite.

The crystalline structures of the ZnS-TiC-C, ZnS-C, and ZnS composites were characterized via XRD analysis (Figure 1a). All the XRD patterns of the as-prepared samples exhibited a series of peaks at ~29°, 33°, 47°, 56°, and 77°, which are designated to the (111), (200), (220), (311), and (331) planes of the cubic ZnS crystalline phase (PDF#05-0566), indicating the successful synthesis of ZnS. For the ZnS-TiC-C composite, two additional XRD peaks were observed at ~39° and ~56°, corresponding to the crystalline structure of the TiC phase (PDF#74-1219), confirming the conductive TiC phase formation after the two-step HEBM. The presence of the conductive TiC phase in the composite after the HEBM agrees well with previous reports [29,32,34,35]. Compared with the ZnS and ZnS-C composites, the ZnS-TiC-C nanocomposite exhibited broader ZnS peaks with lower intensities, suggesting the size reduction of the ZnS crystallites in the ZnS-TiC-C, which is ascribed to the fracturing and deformation of ZnS particles, caused by contact with the mechanically strong TiC phase during the HEBM [36,37]. Although pure Zn, Ti, and S peaks were observed after the first HEBM step (as shown in Figure 1b), the mixture was completely converted into alloy phases, including ZnS, TiC, and amorphous C, after the second HEBM step (Figure 1a). No additional peaks were detected, suggesting there are no impurities in the final product and that the Zn, S, Ti, and C were completely transformed into ZnS and TiC crystallites and amorphous C after the two-step ball-milling process. A schematic of the overall synthesis of the ZnS-TiC-C nanocomposite is presented in Figure 1c. The metastable Zn-S-Ti alloy was formed after the first HEBM process, followed by the formation of a ZnS-TiC-C composite when amorphous C was added to the Zn-S-Ti mixture upon the second HEBM process. The overall reaction for the synthesis of the ZnS-TiC-C nanocomposite is expressed as follows:

(1) $$\mathrm{Zn}+S+\mathrm{Ti}\;\overset{1\mathrm{st}\;\mathrm{HEBM}}{\to }\;\mathrm{ZnS}+\mathrm{Zn}\;\left(\mathrm{not}\;\mathrm{reacted}\right)+S\;\left(\mathrm{not}\;\mathrm{reacted}\right)+\;\mathrm{Ti}\;\left(\mathrm{not}\;\mathrm{reacted}\right)\;\overset{\begin{array}{c} 2\mathrm{nd}\;\mathrm{HEBM}\\ \left(+\mathrm{carbon}\;\mathrm{black}\right) \end{array}}{\to }\;\mathrm{ZnS}-\mathrm{TiC}-C$$

**Figure 2:**
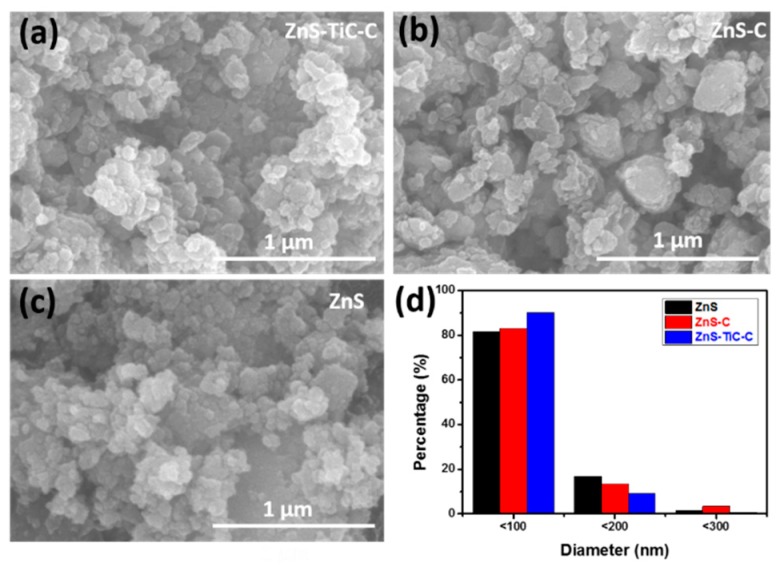
SEM images of the (**a**) ZnS-TiC-C, (**b**) ZnS-C, (**c**) ZnS composites; and (**d**) particle-size distributions of the ZnS, ZnS-C, and ZnS-TiC-C composites.

Figure 2 shows the microscopic morphologies and particle-size distributions of the ZnS-TiC-C, ZnS-C, and ZnS composites. As indicated by the SEM images (Figure 2a–c), the average particle size for all the samples ranged from a few micrometers to hundreds of nanometers. Compared with the ZnS-C and ZnS, the ZnS-TiC-C nanocomposite had a larger percentage of particles with a size of <100 nm (up to 90%, as shown in Figure 2d). The remarkable decrease in the average particle size of the ZnS-TiC-C composite confirmed the continuous fracturing of ZnS particles with the hard and strong TiC phase during the HEBM process [29,32].

**Figure 3:**
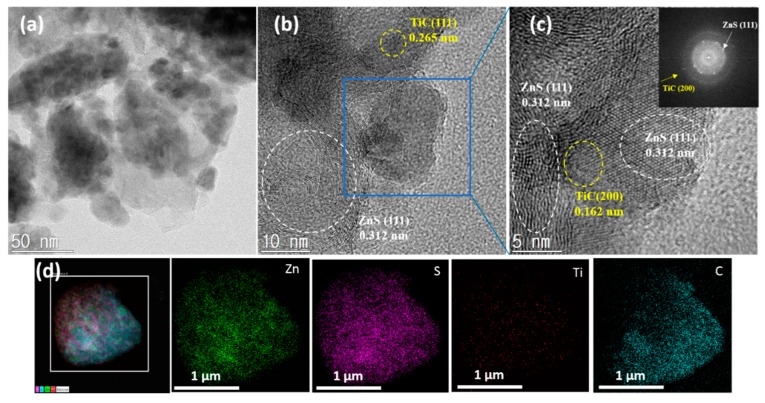
(**a**–**c**) TEM and HRTEM images of the ZnS-TiC-C composite; and (**d**) STEM image with EDS mapping images of the ZnS-TiC-C composite.

The morphology and elemental distribution of the ZnS-TiC-C nanocomposite were analyzed via transmission electron microscopy (TEM), HRTEM, and STEM, together with EDS mapping (Figure 3). The low- and high-resolution TEM images shown in Figure 3a–c reveal the detailed microstructure of the ZnS-TiC-C nanocomposite. The ZnS and TiC nanocrystallites (darker areas of 5–10 nm) were embedded in an amorphous C matrix (brighter areas). The high-resolution TEM images presented in Figure 3b and Figure 3c exhibited two different interplanar spacings (0.312 and 0.162 nm), corresponding to the (111) plane of crystalline ZnS and the (200) plane of crystalline TiC, respectively, in agreement with the XRD results in Figure 1a. Using the selected-area electron diffraction patterns (inset in Figure 3c), the presence of crystalline ZnS (111) and TiC (200) was confirmed, by indexing the interplanar distance and lattice plane. Furthermore, the STEM images and EDS elemental mapping revealed that there was a uniform distribution of nanosized ZnS and TiC throughout the carbon matrix. The results confirmed the small amount of Ti (5.33 wt%) in the ZnS-TiC-C nanocomposite (Figure 3d). The powder morphology is considered to be advantageous for the anode material in LIBs because of the strong buffering effect of the well-mixed TiC-C hybrid matrix against large volume expansions, as well as the enhanced conductivity in the nanocomposite.

**Figure 4:**
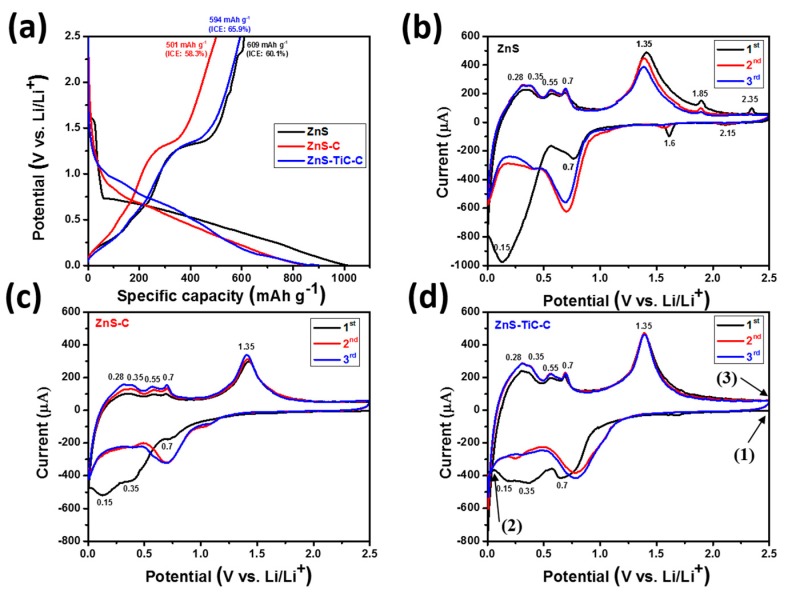
(**a**) Initial charge/discharge profiles and (**b**–**d**) cyclic voltammogram (CV) curves of the ZnS, ZnS-C, and ZnS-TiC-C electrodes.

Galvanostatic charge/discharge measurements were conducted at 0.1 A g^−1^ over the range of 0.01–2.5 V (vs. Li/Li^+^). Figure 4a presents the initial charge/discharge profiles of the ZnS, ZnS-C, and ZnS-TiC-C electrodes. The ZnS and ZnS-C electrodes delivered initial discharge/charge capacities of 1014/609 and 859/501 mA h g^−1^/mA h g^−1^, respectively, corresponding to low initial coulombic efficiencies (ICEs) of ~60% and ~58%, respectively. In comparison, the ZnS-TiC-C electrode exhibited a higher ICE (~66%), as evidenced by the initial discharge and charge capacities of 903 and 594 mA g h^−1^, respectively. The initial capacity loss was primarily caused by the irreversible reaction of active ZnS with Li^+^ ions, and the SEI layer formed on the electrode surface [29,32]. Thus, the ZnS-TiC-C nanocomposite was expected to improve the reversibility of the electrode, because the introduction of the TiC-C phase significantly suppressed the volume change of the active ZnS particles, enhancing the ICE.

Figure 4b–d present the CV curves of the ZnS, ZnSC, and ZnS-TiC-C composites for the initial three cycles, as consistent with previous reports of ZnS-based anode materials [42,43,47]. The CV curve of the ZnS electrode (Figure 4b) was used to investigate the electrochemical reactions between ZnS and Li. During the first discharge process of the ZnS electrode, a small cathodic peak appeared around 0.7 V, which was related to the reduction reaction of ZnS to generate Li_2_S and metallic Zn. The large and broad peak in the voltage range of 0.01–0.4 V corresponds to the further sequential lithiation reactions of Zn to form various Li_x_Zn phases, eventually forming the LiZn phase (Zn → LiZn_4_ → LiZn) in the fully lithiated state [43,44]. The cathodic peaks are partly ascribed to the SEI layer formation on the electrode surface, which was responsible for part of the irreversible capacity. During the first charge process, four anodic peaks were detected in the voltage range of ~0.3–0.7 V, indicating the successive delithiation reactions from LiZn to Zn through several steps ($\mathrm{LiZn}\;\to {\mathrm{Li}}_{2}{\mathrm{Zn}}_{3}\to {\mathrm{LiZn}}_{2}\to {\mathrm{LiZn}}_{4}\to \mathrm{Zn})$ [23,33]. The next oxidation peak was observed at ~1.35 V, corresponding to the recombination of ZnS from Zn and Li_2_S. The basic intercalation reaction of ZnS can be expressed as follows [42,43,44,47]:

(2) $$\mathrm{ZnS}+{\;\mathrm{Li}}^{+}\;+{\;e}^{-}\;\to \mathrm{Zn}+{\;\mathrm{Li}}_{2}S$$

(3) $$\mathrm{Zn}+{\mathrm{xLi}}^{+}+{\;\mathrm{xe}}^{-}\to {\mathrm{Li}}_{x}\mathrm{Zn}\;$$

In the subsequent cycles, the reduction and oxidation peaks almost coincided with each other, suggesting the excellent reversibility of the ZnS-based electrodes. CV behaviors similar to those of the ZnS electrode were observed for the ZnS-C and ZnS-TiC-C electrodes, as shown in Figure 4c,d, except for the additional two pairs of redox peaks at 1.85/1.6 V and 2.35/2.15 V, which may have resulted from the formation of a ZnO impurity in the case of the ZnS electrode [43].

**Figure 5:**
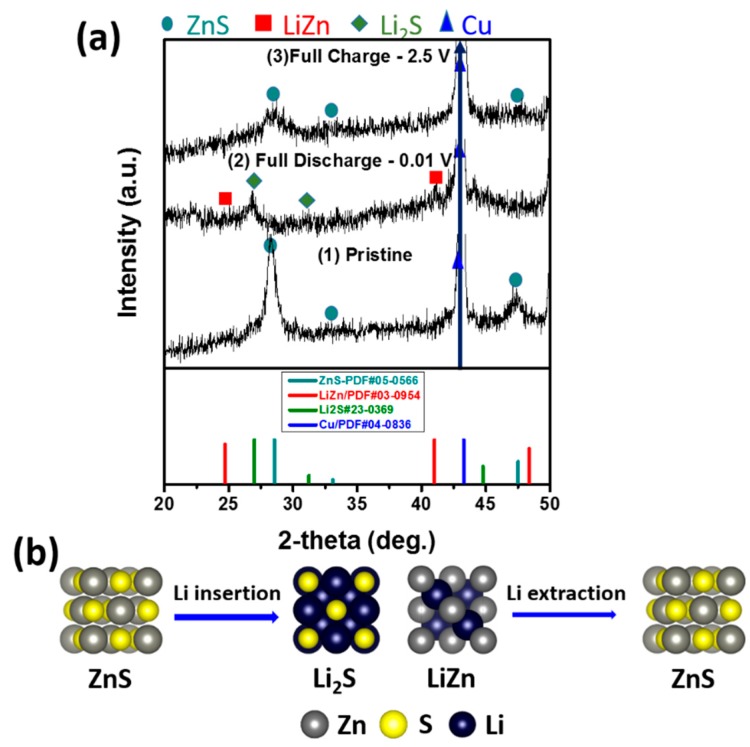
(**a**) Ex-situ XRD patterns for the ZnS-TiC-C electrode after the first charge/discharge cycle and (**b**) schematic of the reaction mechanism.

To elucidate the lithiation/delithiation reaction mechanism of the ZnS-TiC-C electrode during cycling, the ex-situ XRD experiments were performed for the ZnS(80%)-TiC-C electrode (Figure 5a). The three cutoff potentials marked in Figure 4d (see arrows) correspond to the pristine, fully discharged, and charged states of ZnS-TiC-C. Clearly, all the ZnS peaks were observed for the pristine state, in agreement with the XRD results (Figure 1a). After fully discharged to 0.01 V, additional peaks of Li_2_S (~27° and ~31°) and LiZn (~25° and ~41°) were observed, and the ZnS peaks disappeared, indicating that the ZnS phase was completely converted into Li_2_S and LiZn phases. In contrast, at the fully charged state at 2.5 V, all of the LiZn and Li_2_S peaks disappeared, and the ZnS peaks reappeared. This result indicates the reversible lithiation/delithiation of ZnS and suggests that no structural changes occurred during the cycling process. The TiC peaks were not clearly observed in the ex-situ XRD results, owing to the low amplitude of the signal in the measurements, as well as the low TiC content (6.67%) in the ZnS-TiC-C nanocomposite. A schematic of the electrochemical mechanism is presented in Figure 5b.

**Figure 6:**
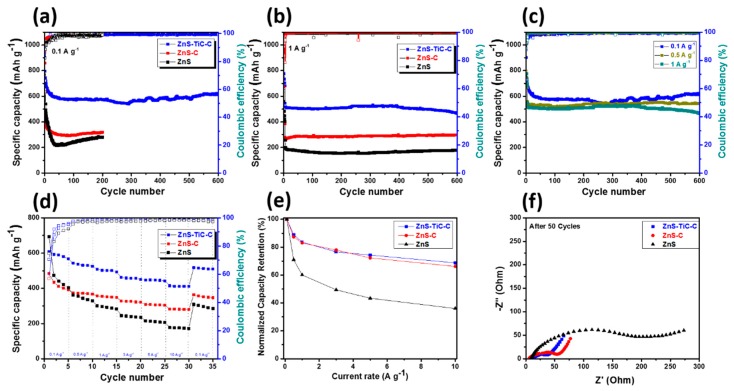
(**a**) Cyclic performance of the ZnS, ZnS-C, and ZnS-TiC-C electrodes at 0.1 A g^−1^; (**b**) long-term cyclic performance of the ZnS, ZnS-C, and ZnS-TiC-C electrodes at 1 A g^−1^; (**c**) comparison of the long-term cyclic performance of the ZnS-TiC-C electrodes at 0.1, 0.5, and 1 A g^−1^; (**d**) rate capability of the ZnS, ZnS-C, and ZnS-TiC-C electrodes; (**e**) normalized capacity retention of the ZnS, ZnS-C, and ZnS–TiC–C electrodes at different current densities ranging from 0.1 to 10 A g^−1^; and (**f**) electrochemical impedance spectra of the ZnS, ZnS-C, and ZnS-TiC-C electrodes after 50 cycles.

Figure 6a compares the cyclic performances and coulombic efficiencies (CEs) of the ZnS-based electrodes at 0.1 A g^−1^ current density. The detailed electrochemical data are presented in Table 1. The ZnS composite delivered a very high initial capacity of ~1014 mA h g^−1^, but the capacity sharply decreased to ~279 mA h g^−1^ after 200 cycles, which is significantly lower than its theoretical value (829 mA h g^−1^) [44]. This is ascribed to the high volume change of active ZnS particles, which were pulverized and delaminated during the cycling. The ZnS-C electrode exhibited a slightly higher reversible capacity and better capacity retention (318 mA h g^−1^ after 200 cycles), mainly owing to the C matrix surrounding the nanosized ZnS, which stabilized the lithiation/delithiation processes. The ZnS-TiC-C nanocomposite electrode exhibited excellent cyclic stability, delivering a highly stable reversible capacity of ~613 mA h g^−1^ over 600 cycles, corresponding to a capacity retention of ~77%, which was significantly higher than that of ZnS-C (~50%) and ZnS (~44%). This superior cyclic performance is attributed to the introduction of the TiC-C phase into the ZnS alloy, which not only increased the conductivity of the composite, but also acted as a reinforcing matrix that mitigated the volume changes and suppressed the aggregation of the ZnS active material upon extended cycling, significantly enhancing the cycling stability of the ZnS-TiC-C nanocomposite [29,32]. The optimum active ZnS content in the composite was determined by measuring the cyclic performance of various ZnS-TiC-C composite electrodes at 0.1 A g^−1^ (Figure S1). Interestingly, all the ZnS–TiC–C composite electrodes exhibited excellent cycling stability over 600 cycles when the ZnS content was varied from 60% to 90%. Among the investigated ZnS-TiC-C nanocomposite electrodes, ZnS(80%)-TiC-C exhibited the best performance, delivering a very stable reversible capacity of ~613 mAh g^−1^ after 600 cycles, with the corresponding stable Coulombic efficiency (CE) of ~99%. The long-term cyclic performance of the ZnS, ZnS-C, and ZnS-TiC-C electrodes were further measured at 1 A g^−1^ (Figure 6b). The ZnS–TiC–C electrode exhibited the best performance, delivering a significantly higher capacity (~467 mA h g^−1^) after 600 cycles than the ZnS-C and ZnS electrodes (~297 and ~179 mA h g^−1^, respectively).

As shown in Figure 6c, although a capacity drop was observed relative to the specific capacity at 0.1 A g^−1^, the cyclic performance of the ZnS(80%)-TiC-C electrode at the high current densities of 0.5 A g^−1^ and 1 A g^−1^ were very stable: the electrode delivered reversible capacities of ~545 mA h g^−1^ at 0.5 A g^−1^ and 467 mA h g^−1^ at 1 A g^−1^ after 600 cycles. The rate capabilities of the ZnS-based electrodes at various current densities in the range of 0.1–10 A g^−1^ were measured and compared, as shown in Figure 6d. Similar to the case of the cyclic performance at 0.1 A g^−1^, among the ZnS-based composite electrodes, the ZnS(80%)-TiC-C nanocomposite exhibited the highest average capacity at all current densities. Specifically, it delivered capacities of ~599, 533, 501, 457, 439, and 412 mA h g^−1^, respectively, at 0.1, 0.5, 1, 3, 5, and 10 A g^−1^ with the corresponding capacity retentions of 100%, 89%, 84%, 76%, 73%, and 69% (relative to the initial capacity measured at 0.1 A g^−1^). In comparison, the ZnS–C and ZnS electrodes exhibited poorer rate capabilities. At the high current density of 10 A g^−1^, they delivered low specific capacities of ~281 and 175 mA h g^−1^ with the corresponding capacity retentions of 66% and 36%, respectively. Furthermore, when the electrodes were returned to its original value of 0.1 A g^−1^, the capacity-retention values were 86%, 83%, and 61% for the ZnS-TiC-C, ZnS-C, and ZnS electrodes, respectively, suggesting the superior rate performance of the ZnS-TiC-C nanocomposite. The normalized capacity retentions in Figure 6e confirm the relatively high capacity retention of the ZnS-TiC-C electrode compared with the ZnS and ZnS-C electrodes. This excellent performance indicates that the introduction of the TiC-C hybrid matrix facilitated Li^+^-ion transport between the active ZnS particles and mitigated the agglomeration and pulverization of the electrode during prolonged cycling [28,29,30,31,32,33]. Figure 6f shows Nyquist plots for the electrodes after 50 cycles. The detailed data for the charge-transfer resistance (R_ct_) and the SEI layer resistance (R_s_) are presented in Table S2. Among the electrodes, ZnS-TiC-C exhibited the best performance, as evidenced by the smallest semicircle and the lowest value of R_ct_ after 50 cycles. In order to investigate the stability of a thin SEI layer during cycling, the EIS measurements were conducted at the fully discharged states of ZnS-TiC-C electrodes for the 1st, 2nd, and 7th cycle. The EIS spectra at the fully discharged state for the 1st cycle is typically different from the ones after the 2nd and 7th cycles, which is consistent with the CV results (Figure 4d). In particular, two semicircles were observed in the EIS spectra after being fully discharged, corresponding to the SEI layer resistance (R_s_) for the first semicircle and the charge-transfer resistance (R_ct_) for the second semicircle. As seen in Figure S2, the EIS spectra of the 2nd and 7th cycles show the similar shape and size for the first semicircle, indicating the resistance of the SEI layer (R_s_) was nearly unchanged during cycling. These results indicate that the introduction of the TiC-C hybrid matrix to active ZnS anode provides effective electron pathways and maintains a stable SEI layer during cycling, thereby improving the interfacial stability and electronic conductivity of the composite. The sheet resistance measurements were further conducted using 4-point probe method (CMT-SR1000 N) to confirm the high conductivity of the ZnS-TiC-C nanocomposite. The slurries of ZnS, ZnS-C, and ZnS-TiC-C were first cast on the Si wafer with the thickness of 30 µm. The sheet resistances (R_sh_) of the ZnS-TiC-C, ZnS-C, and ZnS were measured to be 294 ± 3, 346 ± 8, and 522 ± 46 Ω/sq, respectively. This corresponds to the conductivities (σ = 1/(R_sh_ × t)) of 113 ± 1, 96 ± 2, and 64 ± 6 S/cm, indicating the highest conductivity of ZnS-TiC-C, followed by ZnS-C and ZnS, as shown in Figure S3.

**Figure 7:**
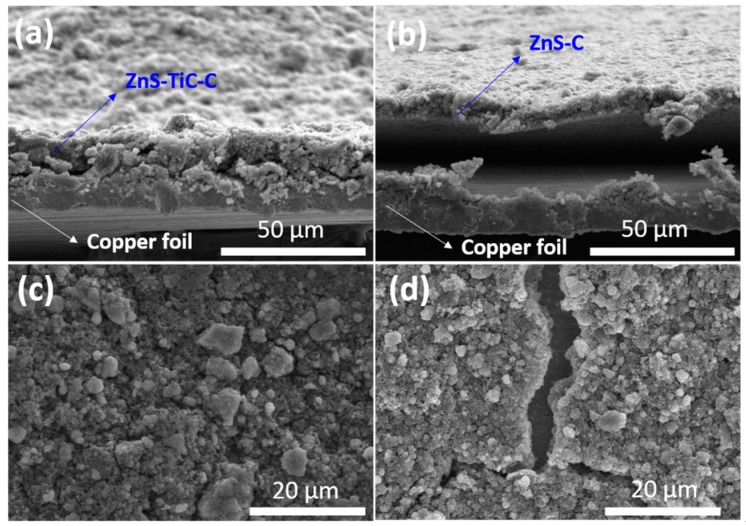
Ex situ SEM images of the (**a**,**c**) ZnS-TiC-C and (**b**,**d**) ZnS-C electrodes after 50 cycles at 0.1 A g^−1^.

The morphologies of the ZnS-TiC-C and ZnS-C electrodes were observed after 50 charge/discharge cycles using ex-situ SEM (Figure 7). The ZnS-C electrode film was partly peeled off from the current collector and exhibited a poor morphology, with significant aggregation, pulverization, and cracks (Figure 7b,d). In contrast, the ZnS-TiC-C electrode exhibited better morphology without any aggregations, delamination or cracks (Figure 7a,c). This result can explain the superior electrochemical performance of ZnS-TiC-C.

**Figure 8:**
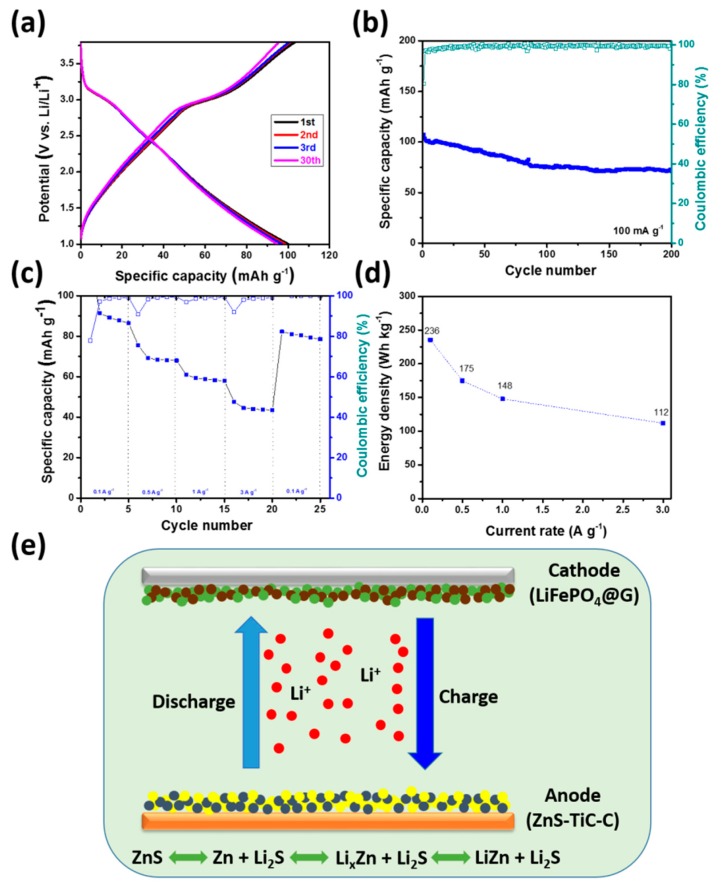
Performance of the full cell using ZnS(80%)-TiC-C as an anode and LiFePO_4_-G as a cathode: (**a**) galvanostatic charge/discharge profiles obtained at 0.1 A g^−1^ over the voltage range of 1–3.8 V; (**b**) cyclic performance at 0.1 A g^−1^; (**c**) rate capability at different current densities ranging from 0.1 to 3 A g^−1^; (**d**) energy density at different current rates of the ZnS-TiC-C//LFP-G full cell; and (**e**) schematic of the ZnS–TiC-C//LFP-G full cell.

To demonstrate the feasibility of the ZnS-TiC-C composite for practical energy-storage applications, LIB full-cell investigations were performed using the ZnS-TiC-C as an anode and the synthesized LiFePO_4_-graphite (LFG-G) as a cathode, as shown in Figure 8. Considering the specific capacity balance, the mass ratio of the cathode to the anode was ~2:1. Figure 8a presents the charge/discharge profiles of the ZnS-TiCC//LFP-G full-cell at 0.1 A g^−1^ in the range of 1.0‒3.8 V. A nominal cell voltage at ~2.5 V was observed during the charge/discharge process, resulting from the distinct reaction potentials between LiFePO_4_ and ZnS. As shown in Figure 8b, the ZnS-TiC-C//LFP-G full cell exhibited a highly stable lifecycle without remarkable capacity fading at 0.1 A g^−1^, delivering a specific capacity of ~71 mA h g^−1^ (calculated using the total mass of the anode and cathode) even after 200 cycles. The full cell exhibited reversible capacities of ~95, 70, 59, and 45 mA h g^−1^, respectively, at 0.1, 0.5, 1, and 3 A g^−1^ (Figure 8c). Although the capacity gradually decreased with the increasing current rate, it recovered to ~81 mA h g^−1^ (corresponding capacity retention of ~86%) when the current density was turned back to its original value of 0.1 A g^−1^. Furthermore, the duration of the capacity at each current density was excellent, indicating the good rate performance of the ZnS-TiC-C nanocomposite as an anode for full-cell applications. The practical energy densities of the ZnS-TiC-C//LFP-G full cell at different current rates were calculated using the working potential (~2.5 V) and the capacity from rate-capability measurements (Figure 8d). The calculated energy densities of the ZnS-TiC-C//LFP-G full cell were 236, 175, 148, and 112 Wh kg^−1^, respectively, at 0.1, 0.5, 1, and 3 A g^−1^ which were significantly higher than the energy densities of the common full cell using Li_4_Ti_5_O_12_ as an anode and LFP as a cathode (~140 Wh kg^−1^ at 0.1 A g^−1^) [52].

## 4. Conclusions

We synthesized a ZnS-TiC-C nanocomposite using a facile, low-cost, and eco-friendly HEBM process, for use as an anode material in LIBs. Structural and morphological characterizations revealed that ZnS nanocrystallites were uniformly dispersed in a multifunctional TiC-C hybrid matrix, resulting in superior electrochemical performance (i.e., stable reversible capacity, exceptional cyclability, and good rate capability) compared with ZnS-C and ZnS composites. The electrochemical performance was remarkably improved by the introduction of TiC to the ZnS-C composites, because the TiC-C buffering matrix offers high electronic conductivity to the active ZnS particles and prevents the high volume change during prolonged cycling. The ZnS-TiC-C electrode delivered a highly reversible capacity (613 mA h g^−1^ over 600 cycles, corresponding to 77% capacity retention) and good rate performance, with ~69% capacity retention at a high current density of 10 A g^−1^. Moreover, the ZnS-TiC-C electrode exhibited very stable long-term cyclic performance even at high current densities (~545 mA h g^−1^ at 0.5 at 0.5 A g^−1^ and 407 mA h g^−1^ and 1.0 A g^−1^, respectively, after 600 cycles). Furthermore, a full cell consisting of an LFP–G cathode and a ZnS-TiC-C anode exhibited very stable cyclic performance without a significant capacity reduction, delivering a specific capacity of 71 mA h g^−1^ after 200 cycles. Thus, the ZnS-TiC-C nanocomposite can be a potential candidate for high-performance LIB anodes.

## Figures and Tables

**Table 1:** Electrochemical data for the ZnS-based electrodes.

Electrode	1st Discharge Capacity (mA h g^−1^)	1st Charge Capacity (mA h g^−1^)	1st Coulombic Efficiency (%)	Capacity Retention (%) (*n*th/2nd Discharge Capacity)
ZnS-TiC–C	903	594	65.8	77.2 (*n* = 600)
ZnS-C	859	501	58.3	49.4 (*n* = 200)
ZnS	1014	609	60.1	44.0 (*n* = 200)

## Supplementary Materials

The following are available online at https://www.mdpi.com/2079-4991/9/9/1221/s1, Figure S1: Cyclic performance of various ZnS–TiC–C electrodes at 0.1 A g^−1^, Figure S2: EIS spectra of ZnS-TiC-C electrodes fully discharged states for 1st, 2nd, and 7th cycle, Figure S3: Conductivities of ZnS-TiC-C, ZnS-C, and ZnS, Table S1: ZnS-based anode materials for LIBs, Table S2. EIS data for ZnS-based electrodes after 50 cycles.
