# Supplementary material for: Enhanced Cycle Stability of Zinc Sulfide Anode for High-Performance Lithium-Ion Storage: Effect of Conductive Hybrid Matrix on Active ZnS
Source: Nanomaterials (Basel). 2019 Aug 29;9(9):1221. doi: 10.3390/nano9091221 (PMC6780193; doi:10.3390/nano9091221)
Supplement: Supplementary file 1 [file nanomaterials-09-01221-s001.pdf]

Supplementary Information

Enhanced Cycle Stability of Zinc Sulfide Anode for High-Performance Lithium-Ion Storage: Effect of Conductive Hybrid Matrix on Active ZnS

Quoc Hanh Nguyen, Taehyun Park and Jaehyun Hur *

Department of Chemical and Biological Engineering, Gachon University, Seongnam-si, Gyeonggi-do 13120, Korea

* Correspondence: jhhur@gachon.ac.kr; Tel: +82-10-9370-3596

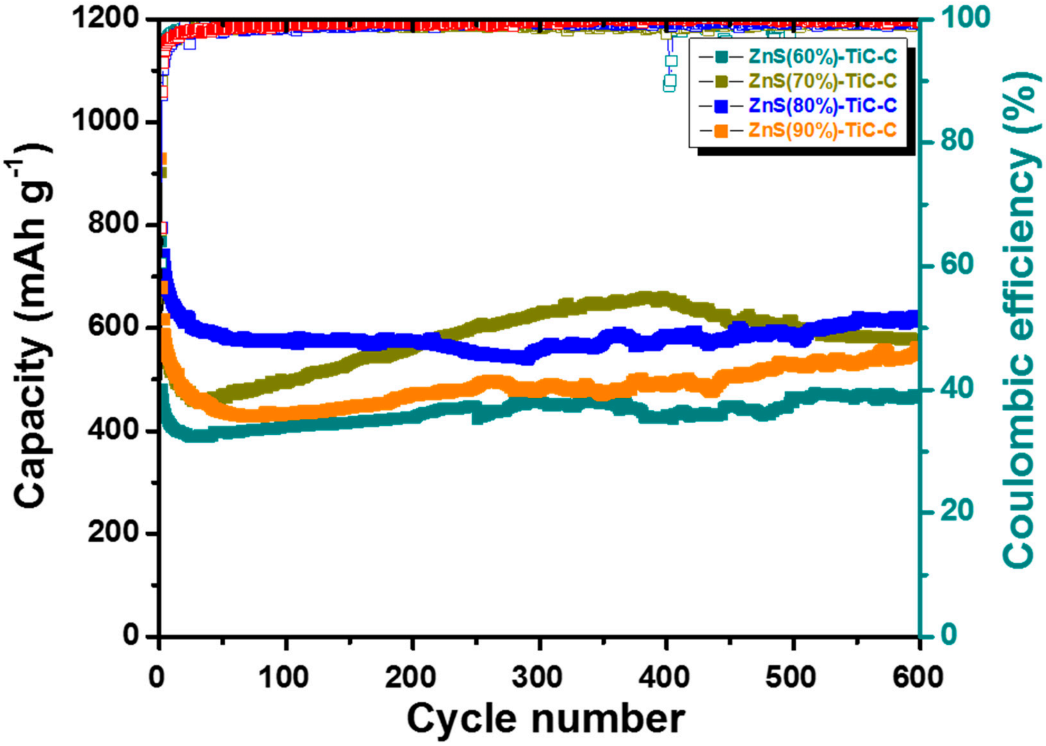

Figure S1. Cyclic performance of various ZnS-TiC-C electrodes at 0.1 A $g^{-1.}$

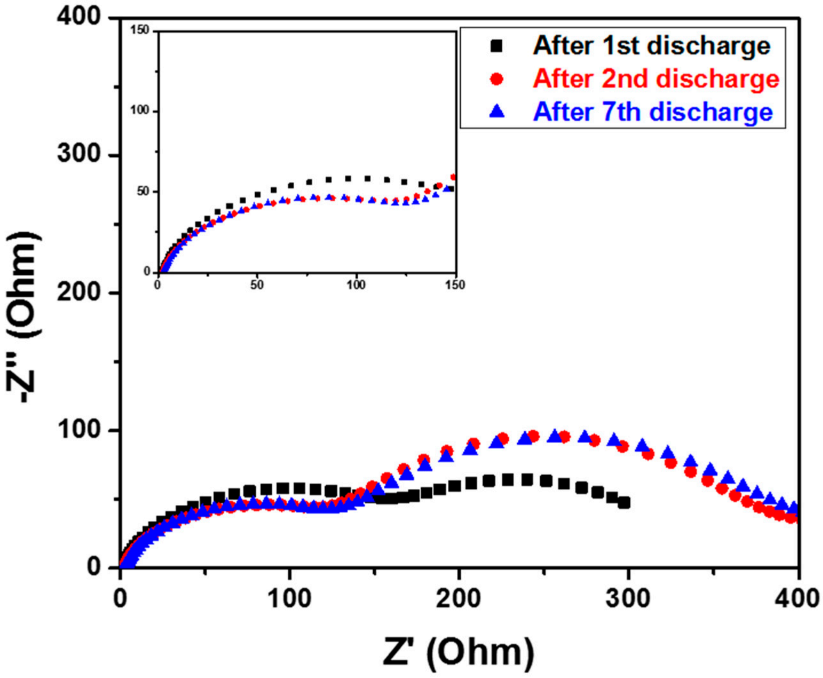

Figure S2. EIS spectra of ZnS-TiC-C electrodes after fully discharged states for 1st, 2nd, and 7th cycle.

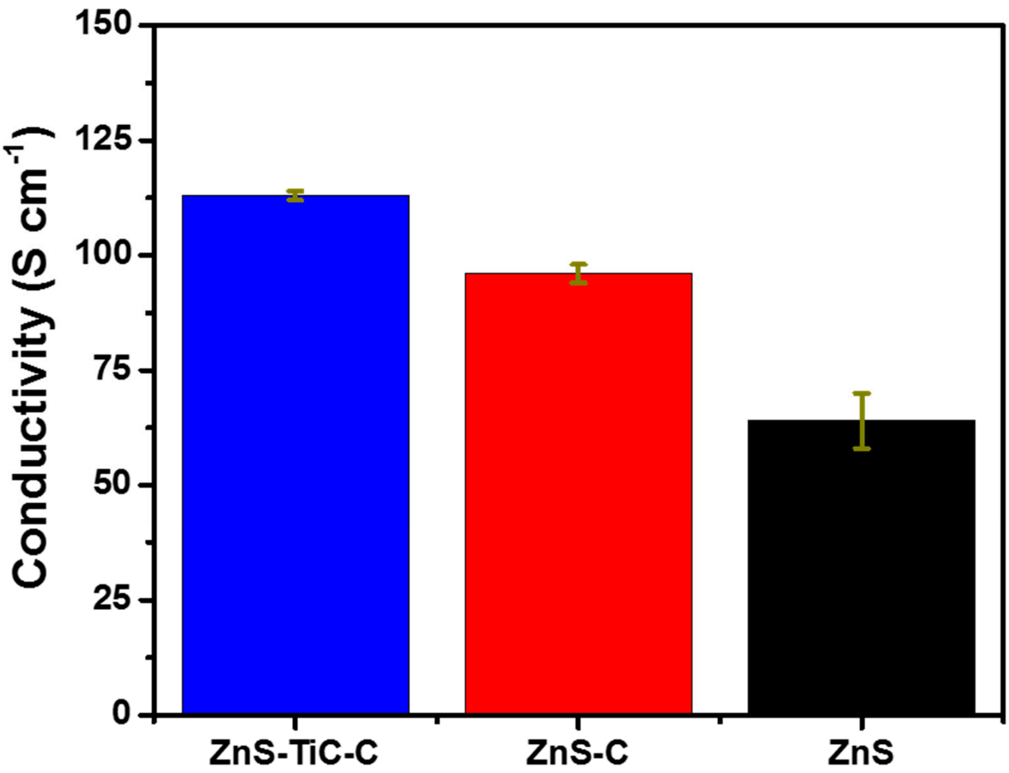

Figure S3. Conductivities of ZnS-TiC-C, ZnS-C, and ZnS.

Table S1. ZnS-based anode materials for LIBs.

Samples	Specific capacity Current density	Cycle number	Rate performance	Ref.
ZnS@C nanoparticles (core-shell)	565 mAh/g (0.1 A/g)	600	363 mAh/g (5 A/g)	[1]
ZnS@C nanocomposite	570 mAh/g (0.1 A/g)	150	405 mAh/g (3 A/g)	[2]
ZnS/graphene composite	570 mAh/g (0.2 A/g)	200	418 mAh/g (1 A/g)	[3]
ZnS@PC (porous carbon)	438 mAh/g (0.1 A/g)	300	180 mAh/g (1 A/g)	[4]
ZnS@C composite	360 mAh/g (0.4 A/g)	300	235 mAh/g (0.8 A/g)	[5]
ZnS@HPC (honeycomb-like porous carbon nanosheets)	408 mAh/g (1 A/g)	200	370 mAh/g (1.5 A/g)	[6]
**ZnS-TiC-C nanocomposite**	**613 mAh/g (0.1 A/g)**	**600**	**412 mAh/g (10 A/g)**	**This work**

Table S2. EIS data for ZnS-based electrodes after 50 cycles.

Samples	After 50 cycles	
	R_s (Ω)	R_{ct} (Ω)
**ZnS-TiC-C**	3.1	37.9
**ZnS-C**	4.9	47.7
**ZnS**	7.9	284.8

References

1. Du, X.; Zhao, H.; Zhang, Z.; Lu, Y.; Gao, C.; Li, Z.; Teng, Y.; Zhao, L.; Świerczek, K. Synthesis of core-shell-like ZnS/C nanocomposite as improved anode material for lithium ion batteries. *Electrochim. Acta* **2017**, *225*, 129-136.
2. Park, A.R.; Jeon, K.J.; Park, C.M. One-pot hydrothermal synthesis of ZnS/C microsphere as an electrode for reversible lithium-storage. *Electrochim. Acta* **2018**, *265*, 107-114.
3. Mao, M.; Jiang, L.; Wu, L.; Zhang, M.; Wang, T. The structure control of ZnS/graphene composites and their excellent properties for lithium-ion batteries. *J. Mater. Chem. A* **2015**, *3*, 13384-13389.
4. Fu, Y.; Zhang, Z.; Yang, X.; Gan, Y.; Chen, W. ZnS nanoparticles embedded in porous carbon matrices as anode materials for lithium ion batteries. *RSC Adv.* **2015**, *5*, 86941-86944.
5. He, L.; Liao, X.Z.; Yang, K.; He, Y.S.; Wen, W.; Ma, Z.F. Electrochemical characteristics and intercalation mechanism of ZnS/C composite as anode active material for lithium-ion batteries. *Electrochim. Acta* **2011**, *56*, 1213-1218.
6. Chen, H.; Zhang, B.; Cao, Y.; Wang, X.; Yao, Y.; Yu, W.; Zheng, J.; Zhang, J.; Tong, H. ZnS nanoparticles embedded in porous honeycomb-like carbon nanosheets as high performance anode material for lithium ion batteries. *Ceram. Int.* **2018**, *44*, 13706-13711.
